# Long-term return to professional wrestling after ankle arthrodesis for end-stage osteoarthritis: a 6-year follow-up case report

**DOI:** 10.1093/jscr/rjaf1090

**Published:** 2026-01-20

**Authors:** Hideaki Fukuda, Tatsuya Takahashi

**Affiliations:** Sports and Joint Center, Inanami Spine and Joint Hospital, 3-17-5 Higashishinagawa, Shinagawa-ku, Tokyo 140-0002, Japan; Department of Orthopaedic, Toyota Memorial Hospital, 1-1 Heiwacho, Toyota city, Aichi 471-8513, Japan

**Keywords:** ankle arthrodesis, post-traumatic osteoarthritis of the ankle, professional wrestler, high demand athlete, return to sport

## Abstract

Post-traumatic ankle osteoarthritis (OA) is a frequent consequence of recurrent injuries among athletes. When conservative management fails, surgical options such as total ankle arthroplasty or arthrodesis are considered. Although ankle arthrodesis (AA) is effective for pain relief, it is generally regarded as incompatible with high-impact sports due to loss of motion and altered biomechanics. We report a 41-year-old professional female wrestler with end-stage post-traumatic AO who underwent arthroscopic AA. She returned to professional competition 6 months postoperatively and continued to perform at an elite level for over 6 years without ankle-related complications. The Japanese Orthopaedic Association score improved from 56.0 preoperatively to 84.7 at the final follow-up. This case demonstrates that with meticulous surgical technique, structured rehabilitation, and sport-specific adaptation, AA can enable selected athletes to achieve sustained high-level function. Careful patient selection and individualized management remain essential.

## Introduction

Post-traumatic ankle osteoarthritis (OA) is a common sequela of recurrent ankle sprains and fractures, which are among the most frequent injuries encountered in athletes [1]. Unlike hip or knee OA, where primary degeneration predominates, ~70%–80% of ankle OA cases are secondary to trauma [2]. Progressive cartilage loss and joint degeneration result in chronic pain, instability, and functional limitation. Once conservative treatment fails, surgical intervention becomes necessary, with total ankle arthroplasty (TAA) and ankle arthrodesis (AA) being the principal options for end-stage disease [3].

AA has long been considered the gold standard for advanced ankle OA, offering reliable pain relief and improved stability through tibiotalar joint fusion [3]. However, this joint-sacrificing procedure inevitably alters biomechanics and may accelerate degeneration of adjacent joints. In contrast, TAA preserves motion but carries risks of implant wear, loosening, and revision surgery, particularly in younger or highly active individuals [4]. Therefore, AA remains widely indicated for patients with high physical demands.

Return to sports after AA is a subject of ongoing debate. Although previous studies have shown significant pain reduction and functional improvement, most patients resume only low- or moderate-impact activities such as walking, swimming, or cycling. High-impact sports that involve explosive movements, rapid directional changes, or axial loading—such as basketball, soccer, or wrestling—are generally discouraged due to the perceived risks of hardware failure, nonunion, or adjacent joint degeneration [5]. Consequently, the consensus remains that return to competitive, high-demand athletics is rarely feasible following ankle fusion.

Here, we describe a rare case of a professional female wrestler with end-stage post-traumatic ankle OA who underwent arthroscopic AA and achieved full return to high-impact competition. Remarkably, she resumed professional wrestling 6 months postoperatively and continued active participation for >6 years without ankle-related complications. This case challenges conventional assumptions and highlights that, under appropriate surgical technique, structured rehabilitation, and sport-specific adaptation, durable athletic function can be achieved even after a joint-sacrificing procedure such as ankle arthrodesis.

## Case report

A 41-year-old professional female wrestler presented with persistent left ankle pain at rest and during activity, accompanied by progressive gait disturbance. She had debuted at age 17 and sustained multiple ankle injuries, including recurrent sprains and fractures, throughout her career. Conservative management included taping, oral and injectable anti-inflammatory medications, and rigid wrestling boots. Despite these measures, her symptoms worsened, and at age 41, she was unable to continue competition due to severe pain and functional limitation.

**Figure 1:**
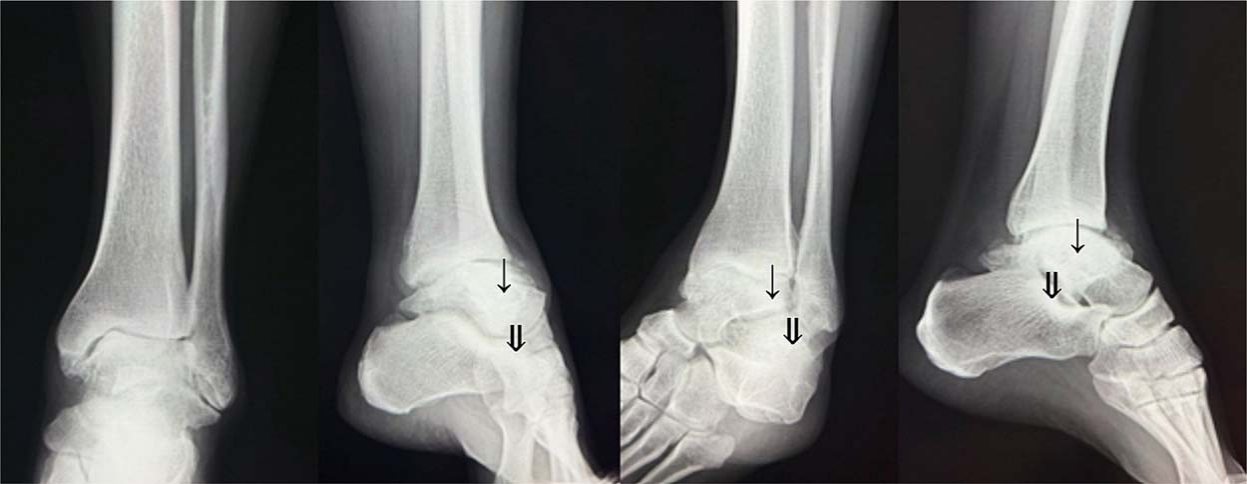
Preoperative radiological imaging: X-ray finding. The patient presented with end-stage osteoarthritis of the tibiotalar joint, characterized by flattening of the joint surface and subluxation of the talus (↓). Although mild arthritic changes were observed in the subtalar joint (⇓), its articular surface remained preserved.

**Figure 2:**
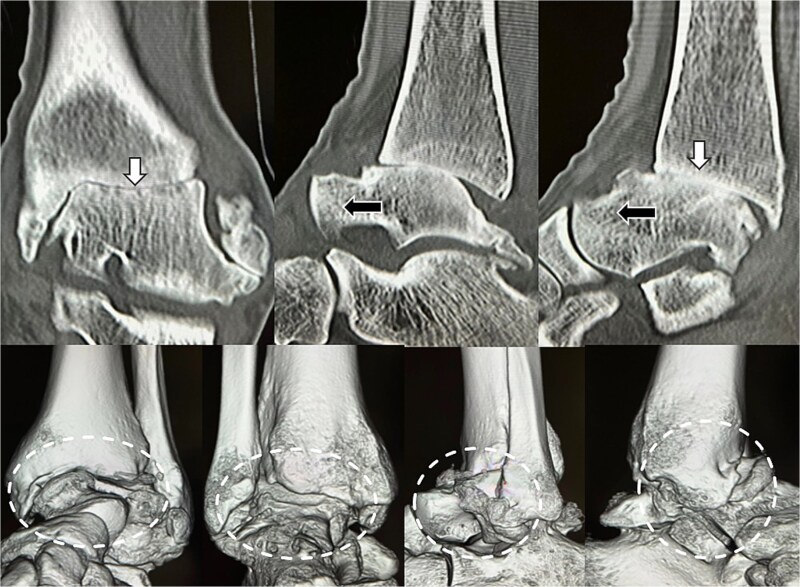
Preoperative radiological imaging: CT finding. The articular surface is completely collapsed and obliterated (1: white arrowhead). The talus is positioned in a subluxated state, and multiple osteophytes are observed on the medial, lateral, and posterior aspects (2–4: black arrowhead / circle with dotted outline). Both radiographs and CT images demonstrated end-stage osteoarthritis of the ankle, characterized by complete collapse of the articular surface and multiple osteophytes along the anterior, lateral, and posterior aspects of the joint (circle with dotted outline).

Preoperative radiographs and computed tomography (CT) demonstrated complete loss of the tibiotalar joint space, talar subluxation, and extensive osteophyte formation consistent with end-stage post-traumatic osteoarthritis (Figs 1 and 2). After informed consent and institutional review board approval (IRB number #20251001), the patient underwent arthroscopic ankle arthrodesis.

**Figure 3:**
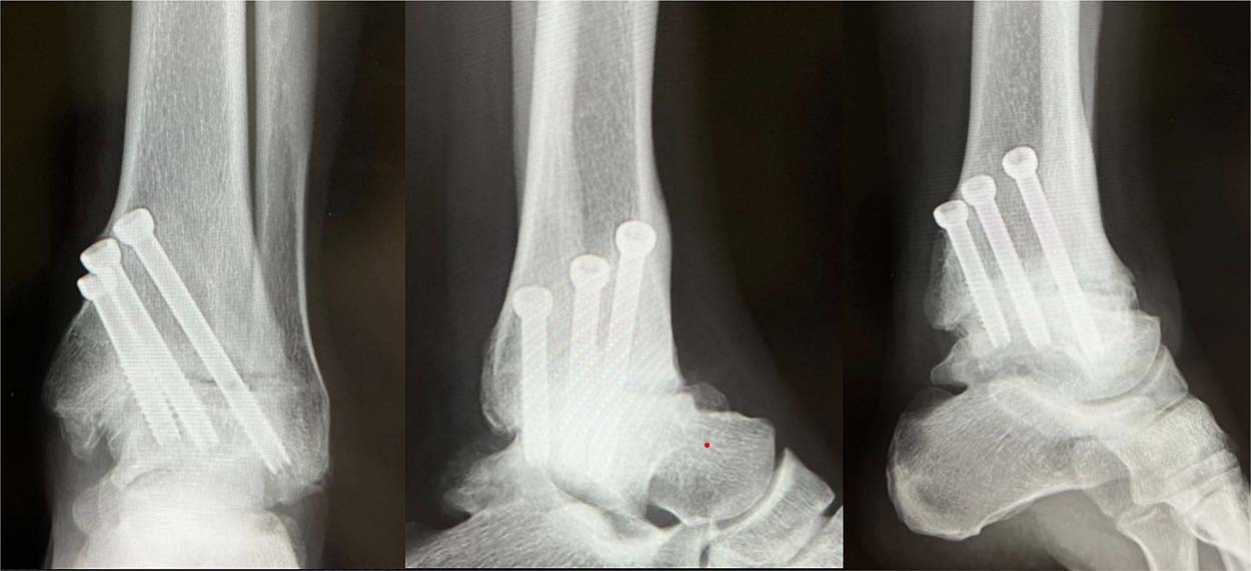
Immediate postoperative radiograph (X-ray). Ankle arthrodesis using three 6.5-mm cannulated cancellous screws (CCS).

Under arthroscopic visualization, inflamed synovial tissue and degenerated cartilage were removed until healthy subchondral bone was exposed. The tibiotalar joint was anatomically reduced and fixed in neutral alignment with axial compression using three 6.5-mm cannulated cancellous screws inserted from the medial malleolus (Fig. 3).

### Postoperative rehabilitation

**Figure 4:**
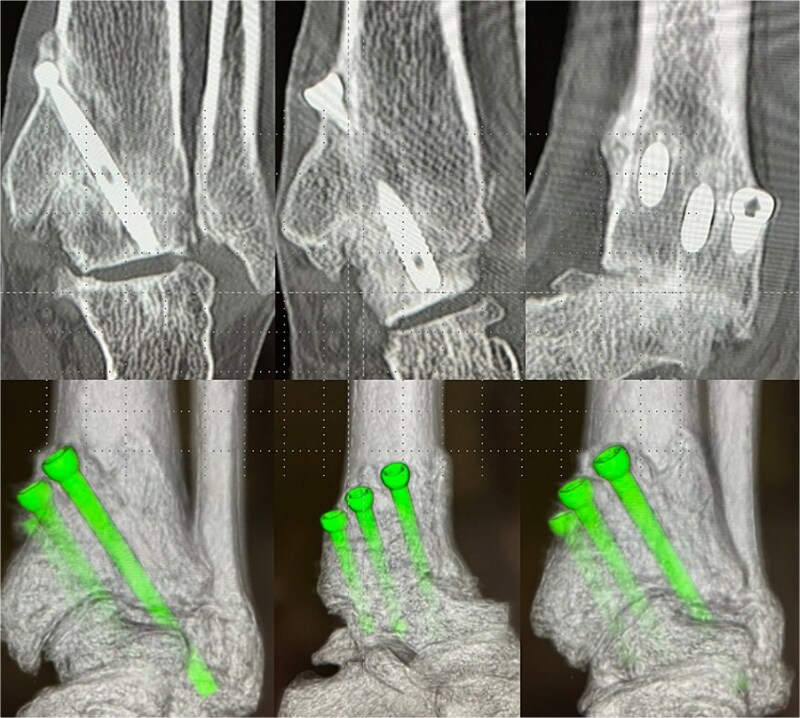
Postoperative-6 months radiological imaging: CT finding. The tibiotalar joint has achieved bony union, and the articular surfaces are anatomically reduced with a horizontal alignment.

For the first four weeks, the patient remained non-weight-bearing in a below-knee cast, performing only toe range-of-motion exercises. Partial weight-bearing was initiated at week 5 and advanced weekly to full weight-bearing by week 8. An ankle-supporting walking boot was used until bone union was confirmed radiographically and by CT at 12 weeks (Fig. 4). At six months postoperatively, she returned to full professional wrestling matches while wearing customized rigid high-cut boots for joint protection.

**Figure 5:**
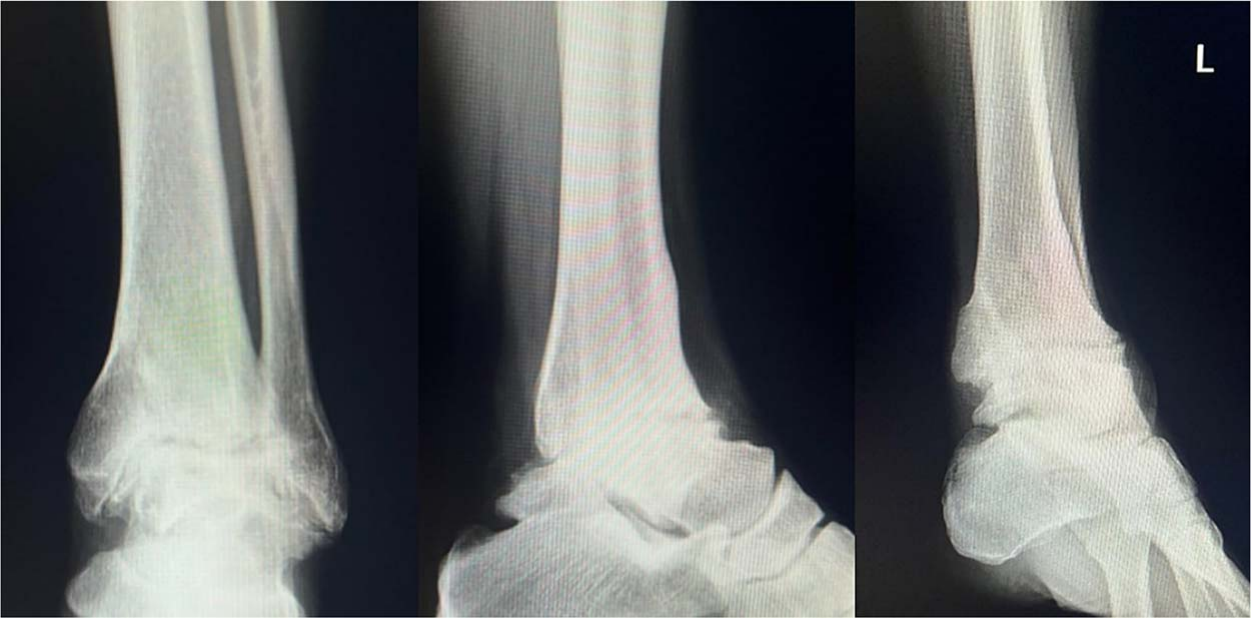
Plain radiograph after hardware removal. At 1.5 years after surgery, the patient underwent hardware removal. Although the osteoarthritic changes in the subtalar joint have slightly progressed, radiographic evaluation confirmed solid osseous union at the tibiotalar joint.

At 1.5 years, hardware removal was performed, with imaging confirming solid osseous union (Fig. 5). The Japanese Orthopaedic Association (JOA) score improved from 56.0 preoperatively to 81.4 at one year and 84.7 at the final 6.5-year follow-up. She resumed 60–70 matches per year without ankle pain, swelling, or motion-related discomfort. Her retirement after 6.5 years was unrelated to the ankle, resulting instead from cervical spine trauma and bilateral knee degeneration. Throughout follow-up, she remained free of ankle-related complications or functional restrictions.

The JOA score is a standardized clinical assessment tool widely used in Japan to evaluate foot and ankle conditions. It consists of four domains—pain, function, walking ability, and range of motion—yielding a maximum total score of 100 points. Higher scores represent better clinical status. Because the JOA score is not widely utilized outside Japan, we included this description to enhance international readers’ understanding of the metric.

## Discussion

AA remains a well-established procedure for end-stage ankle osteoarthritis, consistently providing pain relief and mechanical stability [6, 7]. However, the resulting loss of tibiotalar motion inevitably alters lower-limb biomechanics and has raised concerns regarding long-term degeneration of adjacent joints, particularly the subtalar and midfoot joints [8]. Consequently, participation in high-impact sports following ankle fusion is uncommon, and most patients are typically limited to low- or moderate-impact activities such as walking, swimming, or cycling [8, 9].

The present case is exceptional in that an elite professional wrestler returned to full competitive participation only 6 months after arthroscopic AA and continued to perform at a professional level for >6 years without ankle-related complications. To our knowledge, only a limited number of reports have documented sustained high-impact athletic participation following AA [10, 11]. This outcome suggests that, under specific conditions, ankle fusion does not necessarily preclude long-term engagement in high-demand sports.

The use of an arthroscopic technique may have contributed to the favourable outcome observed in this case. Arthroscopic AA has been reported to preserve periarticular soft tissues, minimize surgical trauma, and reduce disruption of surrounding musculature and vascular supply when compared with open fusion techniques. These advantages may facilitate earlier rehabilitation and promote reliable osseous union, which is particularly beneficial for athletes who require rapid functional recovery. Such factors may partly explain the patient’s ability to resume professional wrestling at 6 months postoperatively.

Professional wrestling involves repetitive axial loading, impact forces, and rotational stresses during takedowns, jumps, and landings. However, many wrestling maneuvers rely less on sagittal-plane ankle motion than sports such as sprinting or soccer. In this context, stable tibiotalar fusion combined with preserved subtalar joint compensation and adequate proximal joint strength may allow functional adaptation. The patient’s ability to modify movement patterns and effectively distribute mechanical loads across the kinetic chain likely played an important role in maintaining long-term performance.

Although AA can increase mechanical loading on the subtalar and midfoot joints, this patient did not develop adjacent joint degeneration during the 6-year postoperative period. Possible contributing factors include achievement of appropriate alignment at the time of fusion, strict postoperative muscle conditioning, and consistent use of customized rigid, cushioned wrestling boots and insoles during both training and competition. These elements may have mitigated excessive stress on adjacent joints and contributed to the durable clinical outcome.

Nevertheless, the findings of this case should be interpreted with caution. Altered biomechanics following AA may predispose patients to progressive degeneration of adjacent joints over time, and such risks cannot be fully assessed based on a single case. Furthermore, comparative studies evaluating return-to-sport rates after AA and TAA report overall return rates of ~60%, with high-impact sports being underrepresented in most cohorts [12, 13]. Therefore, AA should not be routinely recommended for high-demand athletes.

Patient selection remains a critical factor when considering AA in athletes. Favorable conditions in this case included end-stage pain refractory to conservative treatment, preserved subtalar joint integrity, high motivation for return to sport, access to structured rehabilitation, and availability of sport-specific protective footwear. Without these factors, similar outcomes may not be achievable. Thorough preoperative counseling and individualized treatment planning are therefore essential.

From a clinical perspective, this case provides insight into the potential upper limits of functional recovery following AA. While large cohort studies emphasize average postoperative outcomes, individual cases such as this highlight that exceptional long-term function can be achieved under optimal surgical, rehabilitative, and biomechanical conditions. This report may assist surgeons in shared decision-making discussions with elite athletes who face limited surgical options for end-stage ankle osteoarthritis.

## Conclusion

Arthroscopic AA can provide durable pain relief and functional restoration even in elite athletes with end-stage post-traumatic osteoarthritis. This case demonstrates that with careful surgical execution, progressive rehabilitation, and sport-specific adaptation, high-level professional activity can be sustained long-term. Although such outcomes are rare, they emphasize the need for individualized management and close follow-up in highly active patients.

## References

[1] Valderrabano V, Horisberger M, Russell I, et al. Etiology of ankle osteoarthritis. Clin Orthop Relat Res 2009;467:1800–6. 10.1007/s11999-008-0543-6 18830791 PMC2690733

[2] Thomas RH, Daniels TR. Ankle arthritis. J Bone Joint Surg Am 2003;85:923–36. 10.2106/00004623-200305000-00026 12728047

[3] Haddad SL, Coetzee JC, Estok R, et al. Intermediate and long-term outcomes of ankle arthrodesis and ankle arthroplasty: a systematic review. J Bone Joint Surg Am 2007;89:1899–905. 10.2106/JBJS.F.01149 17768184

[4] Dalat F, Trouillet F, Fessy M, et al. Comparison of quality of life following total ankle arthroplasty and ankle arthrodesis: retrospective study of 54 cases. Orthop Traumatol Surg Res 2014;100:761–6. 10.1016/j.otsr.2014.07.018 25306302

[5] Schuh R, Hofstaetter J, Krismer M, et al. Total ankle arthroplasty versus ankle arthrodesis: comparison of sports, recreational activities and functional outcome. Int Orthop 2012;36:1207–14. 10.1007/s00264-011-1455-8 22173565 PMC3353091

[6] Johns WL, Sowers CB, Walley KC, et al. Return to sports and activity after total ankle arthroplasty and arthrodesis: a systematic review. Foot Ankle Int 2020;41:916–29. 10.1177/1071100720927706 32501110

[7] Chuckpaiwong B, Reingrittha P, Harnroongroj T, et al. Sport and exercise activity after isolated ankle arthrodesis for advanced-stage ankle osteoarthritis: a single-center retrospective analysis. Foot Ankle Orthop 2023;8:1–9. 10.1177/24730114231177310 PMC10262617 37325694

[8] Santini F, Müller G, Rossi P. Sports activity with ankle osteoarthritis and total ankle arthroplasty: a 103-patient prospective study. J Orthop Res 2024;13:345–52. 10.3390/jcm13237099

[9] Leucht AK, Veljkovic A. Arthroscopic ankle arthrodesis. Foot Ankle Clin 2022;27:175–97. 10.1016/j.fcl.2021.11.008 35219365

[10] Raufi MY . Ankle arthrodesis revisited: a systematic review of techniques, outcomes, and complications. Cureus 2025;17:e86836. 10.7759/cureus.86836 40718270 PMC12296955

[11] Kerkhoff YRA, Keijsers NLW, Louwerens JWK. Sports participation, functional outcome, and complications after ankle arthrodesis: mid-term follow-up. Foot Ankle Int 2017;38:1085–91. 10.1177/1071100717717221 28708946

[12] Vertullo CJ, Nunley JA. Participation in sports after arthrodesis of the foot or ankle. Foot Ankle Int 2002;23:625–8. 10.1177/107110070202300707 12146773

[13] Hanna M, Whicker EA, Traub B, et al. Sport activity levels following ankle fusion. Int Orthop 2021;45:2347–54. 10.1007/s00264-021-05100-7 34228148

